# A combination of flexible and rigid bronchoscopy in the successful removal of a residual fish bone from a peripheral bronchus: A case report

**DOI:** 10.3389/fped.2023.1114043

**Published:** 2023-02-21

**Authors:** Hanqing Shao, Shuxian Li, Jing He, Lei Wu, Zhimin Chen

**Affiliations:** ^1^ Department of Pulmonology, Children’s Hospital, Zhejiang University School of Medicine, National Clinical Research Center for Child Health, Hangzhou, China; ^2^ Department of Endoscopy Center, Children’s Hospital, Zhejiang University School of Medicine, National Clinical Research Center for Child Health, Hangzhou, China

**Keywords:** flexible bronchoscopy, rigid bronchoscopy, foreign body, inhalation, case report

## Abstract

Although rigid bronchoscopy remains the gold standard for the management of foreign body (FB) inhalation, sometimes it still misses residual FBs. Inhalation of sharp FBs by infants is an uncommon but hazardous occurrence, which presents a significant challenge and demands expertise in therapeutic bronchoscopy. Particularly, residual sharp FBs in the peripheral tracheobronchial tree may pose challenging management problems for bronchoscopists. Herein, we describe the case of 1-year-old girl, who presented with persistent atelectasis in the left lower lobe for 20 days without responding to antibiotic therapy after removal of fish bone by rigid bronchoscopy at local hospital. Flexible bronchoscopy at our department showed a residual fish bone in the outer basal segment of the left lower lobe. A combined flexible and rigid bronchoscopy was then applied, and a fish bone measuring 1.5 cm in length was extracted on multiple attempts without any complications. Thus, our reports demonstrated that removal of challenging residual sharp FBs in the distal airways is possible with the aid of combined flexible and rigid bronchoscopy by an experienced multidisciplinary team. Additionally, a physician should pay special attention to abnormal chest images after removal of FBs.

## Introduction

Pediatric foreign body (FB) aspiration into the tracheobronchial tree is a common cause of respiratory problems, particularly among children younger than 3 years (1). Although it may lead to obstruction and various long-term respiratory consequences (e.g., recurrent pneumonia, atelectasis, bronchiectasis and pulmonary abscess), most FBs are located in the central airway and removed smoothly with either a flexible or a rigid bronchoscope (1). Still, 1%–18% patients may have residual FBs after initial bronchoscopy (2–4), which mainly lodges in the peripheral airway due to small size, progressive migration, repeated attempts at removal, mucosal damage caused by unsuccessful attempted removal, or chronic FB reaction with hypertrophied endobronchial mucosa. These situations present a significant challenge for endoscopists to remove such a peripherally residual impacted object without resorting to open thoracotomy or segmental pulmonary resection, especially for sharp and penetrating objects, which may lead to further mucosal damage and perforation of tracheobronchial tree.

Rigid bronchoscopy under general anesthesia is widely accepted as the intervention of choice for FB extraction in children (5). However, rigid bronchoscopy-related main complications (e.g., bronchospasm, desaturation, and trauma to the respiratory tract with bleeding or edema) occurring in 8%–17% of cases and rare complications (e.g., pneumothorax/mediastinum, need for tracheotomy, cardiac arrest, and even death) were described in literature (6, 7). In recent years, flexible bronchoscopy has increasingly been applied for FB removal with shorter procedure time and minimal complication rate (8). Indeed, different endoscopic centers have different preferences for the procedures for removal of the aspirated FBs (9–14). Some centers perform flexible bronchoscopies in cases of FB aspiration (9, 10, 13), while other centers use rigid bronchoscopy as an initial procedure (11, 12, 14). Nevertheless, there is a general agreement that flexible bronchoscopy should be performed for removal of inhaled FBs with the backup of rigid bronchoscopy (7, 13).

Herein, we describe a 1-year-old girl wherein a residual fish bone lodged in the distal airway in the outer basal segment of the left lower lobe has been successfully managed by the utility of flexible bronchoscopy and rigid bronchoscopy without the necessity for thoracotomy or pulmonary resection. We expect that our study can attribute to the management of some special cases of residual sharp and pointed objects in the future and discuss the advantages of the combination of flexible and rigid bronchoscopy over either of the scopes used alone.

## Case presentation

**Figure 1:**
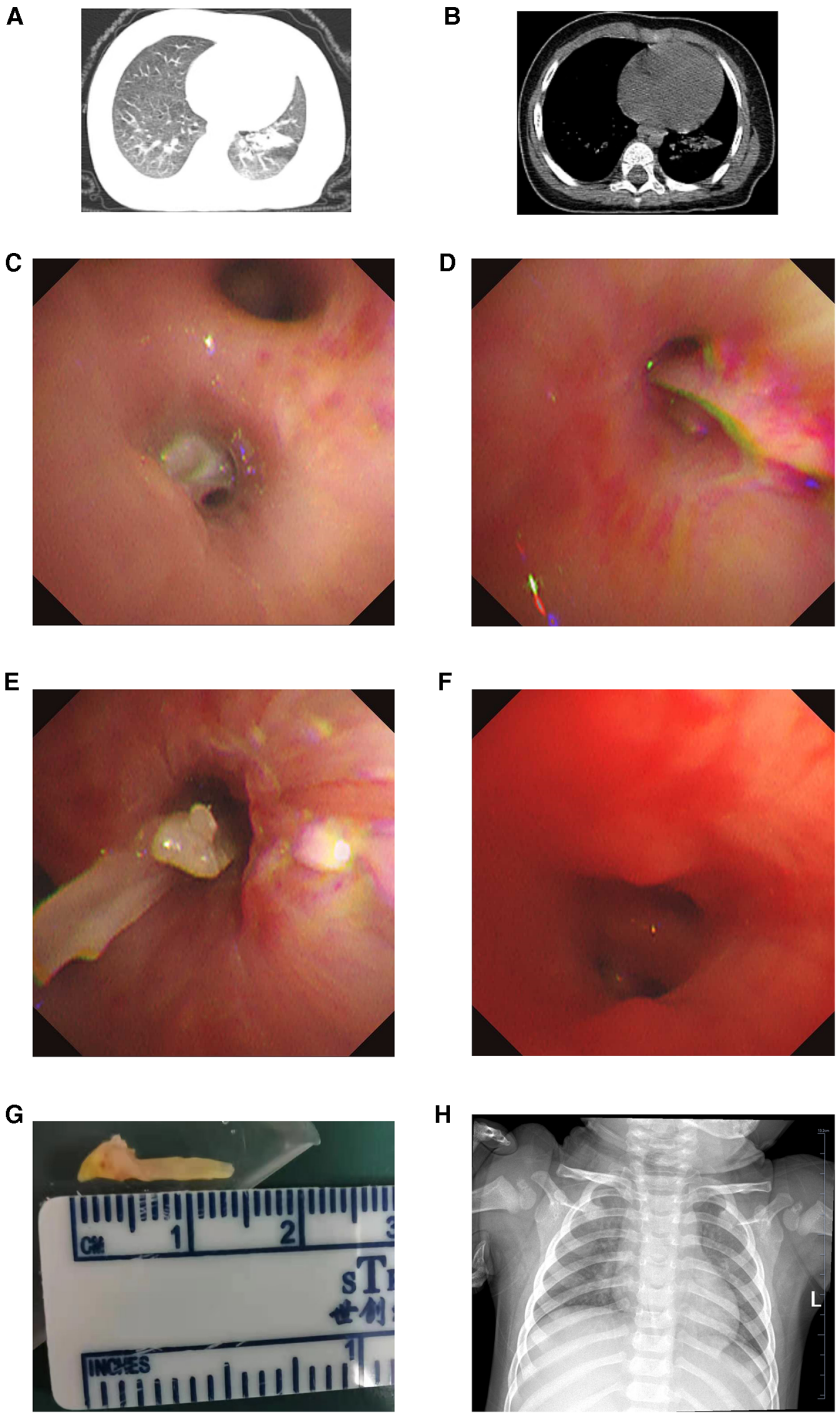
Lung (**A**) and mediastinum (**B**) window of chest CT showing atelectasis in the left lower lobe; (**C**) under flexible bronchoscopy, residual FB lodged in the outer basal segment of the left lower lobe bronchus prior to extraction; (**D,E**) residual fish bone pulled by grasping forceps through flexible bronchoscope; (**F**) the aspect of the outer basal segment of the left lower lobe bronchus after FB removal; (**G**) the residual fish bone measuring 1.5 cm in length; (**H**) the aspect of chest x-ray on discharging. CT, computed tomography; FB, foreign body.

A 1-year-old female was transferred to our department for partially persistent atelectasis in the left lower lobe for 20 days without response to antibiotic therapy from a local hospital. On admission, her parents complained the little girl had recurrent fever and cough over the past 4 months, and treated as pneumonia. One month ago, chest computed tomography (CT) revealed FB in the left main bronchus. Then she underwent rigid bronchoscopy immediately, and a fish bone was removed successfully at a local hospital. Although she remained asymptomatic thereafter, two repeated chest CT after initial bronchoscopy showed partially persistent atelectasis in the left lower lobe without any improvement following antibiotic treatment for about 2 weeks (Figures 1A,B). At the time of presentation, her vital signs were within normal ranges and she had clear air entry on chest auscultation. According to the above **information**, the patient was scheduled for flexible bronchoscopy for disclosing the suspected causes (e.g., retained FB fragments, mucus plug, and granulation tissue). Bronchoscopic examination revealed a fish bone lodged in the outer basal segment of the left lower lobe bronchus (Figure 1C). Considering FB lodgment at the distal airway, flexible bronchoscopy was first recommended for retrieval with the possible need for thoracotomy if bronchoscopy failed. Risks, benefits, and expected complications were explained to her guardians at the same time. Prior to proceeding with thoracotomy, her parents also preferred to choose bronchoscopy as a less invasive procedure.

Grasping forceps was then inserted through the working channel of the flexible bronchoscope, attempting to grasp the fish bone, and the object was pulled in the axis of the airway to prevent it from scratching or getting stuck in the bronchial wall under general anesthesia (Figures 1D–F). The fish bone was successfully moved to the trachea on multiple attempts, but it failed to pass through the glottis with the help of the flexible bronchoscope. Rigid bronchoscopy was carried out immediately, through which the fish bone was withdrawn through the cords and removed from the oropharynx successfully. No complications (e.g., bronchospasm, hemorrhage) were encountered during the procedure. The entire operation under the bronchoscopy lasted for about 1 h. The FB was a sharp and hard fish bone about 1.5 cm in length (Figure 1G). The postoperative period was uneventful and the child was discharged on day 3 with a normal chest film (Figure 1H).

## Discussion

We present a case of a residual sharp distal airway FB, posing a great challenge and requiring ingenuity for its successful removal. Fortunately, the peripheral pointed retained fish bone was successfully withdrawn by the use of grasping forceps through flexible bronchoscopy along with rigid bronchoscopy, ultimately obviating the need for more invasive surgeries (e.g., thoracotomy and bronchotomy). In our case, switching from flexible bronchoscopy to rigid bronchoscopy timely (due to unable to pass through the vocal cords) during the same procedure was the key to successfully removing the FB. Our report highlights that flexible bronchoscopy is a feasible option for FB removal from the distal sharp airway on the available support of rigid bronchoscopy or surgical extraction. It also emphasizes the need for bronchoscopists to be facile with both rigid and flexible endoscopic technique.

Nowadays, flexible endoscopy and rigid endoscopy are both widely used and the chosen method for FB removal differs in different parts of the world (1, 4). Still, location, size, consistency of inhaled FBs, the nature of the FB, its prolonged presence in the tracheobronchial tree, and the history of previous bronchoscopy remain important problems to be considered when choosing methods for removing such objects. Although rigid bronchoscopy allows better transfer and control of force in extracting hard and impacted FBs and can reach the right main and left main bronchus, it cannot reach the more distal branches. As a complement, flexible bronchoscopy allows better visualization of distal airways, especially to reach right upper lobe and basal segments of lower lobe bronchi difficult to access through a rigid bronchoscope. In other words, rigid bronchoscopy is insufficient for FB aspiration running distally, while flexible bronchoscopy enhances visualization and provides excellent exposure utilizing its suction, irrigation, and air insufflation capabilities (8). Undoubtedly, the combination of flexible bronchoscopy and rigid bronchoscopy in our case not only provided excellent visualization and maneuverability but also provided an alternative approach in the removal of a difficult aspirated FB ultimately avoiding open surgical removal. In line with our case, Eyekpegha et al. described a 6-year-old boy who had a history suggestive of an aspirated base cap of a pen despite of two sessions of rigid bronchoscopy and a session of flexible bronchoscopy at three different hospitals, which failed to show the FB. The FB was finally demonstrated on a chest CT image and retrieved by combined rigid and flexible bronchoscopy (15). Similarly, Ruegemer and Perkins reported an 8-year-old male who aspirated a “ball bearing” in right lower lobe bronchus, which failed to be retrieved on two rigid bronchoscopic removal attempts using various instruments, including optical FB forceps, ball bearing forceps, Segura wire basket, rigid FB basket, and Fogarty catheter. After steroid treatments for 48 h, it was successfully extracted utilizing a four wire helical basket inserted through the operating channel of a flexible bronchoscope, which was inserted through a rigid bronchoscope (16). Altogether, these data indicate that rigid and flexible bronchoscopes are complementary tools to each other, especially in complex cases, and the choice of the removal technique should be individualized in different clinical scenarios.

Residual FBs can cause local mechanical effects and mucosal inflammation, and may lead to serious complications, such as recurrent pulmonary infection, atelectasis, bronchiectasis, asthma, lung collapse, empyema, lung abscess, and bronchial fistula (17). Undoubtedly, the prolonged atelectasis in our case was related to both the inflammatory process and local mechanical obstruction initiated by the retained fish bone, highlighting the importance of the clinical history in the diagnosis of residual FBs and the special attention of abnormal chest imagines after removal of FB. Likewise, Xu et al. demonstrated 31 (2.7%) of 1,130 children with residual airway FBs after rigid bronchoscopy confirmed by fiber-optic bronchoscopy (18). Shin et al. reported incomplete removal of an FB at the initial bronchoscopy that occurred in 2 (2.2%) of 92 patients causing migration of the FB fragment into the more distal bronchial tree at the second bronchoscopy (19). Notably, compared with other centers where rigid bronchoscopy was performed without the preceding flexible procedure and showing a wide range of 16%–57% rigid bronchoscopy-negative rate (6), Mansour and Elias reported reduced negative rigid bronchoscopy rate as low as 15% by introducing flexible bronchoscopy as a diagnostic tool prior to performing rigid bronchoscopy in FB aspiration management (9). Altogether, it is crucial for the bronchoscopist to perform a thorough survey of the airway to evaluate for any missed FBs after the removal of any FB.

Inhalation of sharp objects may cause life-threatening complications (e.g., extraluminal migration, perforation of the tracheobronchial tree, bronchial rupture, peripheral migration, pneumothorax, and pericardial tamponade) (20), resulting in their removal being specifically challenging. In the retrospective study conducted by Kaptanoglu et al., FB-related complications were encountered in 4% (13/332) patients, among which sharp pins were responsible for the majority of complications in 69% (9/13) cases (20). However, Ludemann and Riding reported that none presented with pneumothorax or pneumomediastinum among seven adolescents who had aspirated sharp, metallic foreign bodies, and only the patient whose diagnosis was delayed for over a year presented with purulent bronchitis and mild hemoptysis (21). Actually, the accurate incidence of sharp object-related complications is not known due to various reasons, such as the uncommon occurrence of sharp objects aspiration, lifestyle and eating habits in different geographical regions, duration of FB lodgment, and limited studies in the literature. Although the method to remove FBs mainly depends on the type and size of FBs, the time and position of incarceration, the state of patients, and the habits of operators, we propose that a patient with challenging FB inhalation should be referred to a specialized center where both rigid and flexible bronchoscopy can be performed by the same operator. Nonetheless, timely retrieval of sharp objects is imperative and needs to be performed with utmost care. In addition, consultation with pediatric surgery should be performed regarding the possibility of open surgical removal. Multidisciplinary cooperation (e.g., pulmonology, endoscopy, anesthesiology, thoracic surgery) is also essential for unexpected complications (e.g., bronchospasm, pneumothorax, failure of bronchoscopic removal) and likely to improve the success of these procedures for optimal outcomes in challenging FB removal circumstances. Above all, preventive measures including food safety education for parents and pediatricians, public awareness on children, correct execution of the Heimlich maneuver, and cardiopulmonary resuscitation are of great importance for reducing incidence and severity of the airway FB aspiration (22).

The FB residue of our case after initial bronchoscopy may be attributed to several factors, such as the doctor's clinical experience, operating skills, and incomplete exploration of the airway. Indeed, the quality of bronchoscopic interventions remains very variable among doctors, driving practical approaches to be developed to improve this variation among specialists. In this golden age of rapid advances in artificial intelligence (AI), the combination of AI and medicine poses a profound effect on every aspect of healthcare (23) with significant progress in image classification, segmentation, and object detection (24, 25), which shed light on the difficult FB management by bronchoscopy. For instance, Li et al. built a bronchoscopy quality-control system based on AI and showed that the supplemental application of the AI system could reduce the differences in the endoscopic skills of doctors with different levels of experience (26). However, at present, only limited studies have been taken in the application of AI to bronchoscopy (Supplementary Table S1) (26–31), most of which are mainly focused on recognizing tumors (27, 28, 31). Particularly, there is a research gap in the application of AI in complicated FB aspiration. In the near future, the AI system is expected to improve airway management by monitoring the blind spot rate during bronchoscopy, helping bronchoscopists in the identification of key anatomy in real time, identifying retained FBs, and thus improving the quality of bronchoscopic interventions.

In conclusion, our case illustrated the successful retrieval of a distal residual sharp FB by the use of flexible along with rigid bronchoscopic techniques as an optimized treatment without any complications. Both flexible and rigid bronchoscopies are practical therapeutic interventions for the challenging removal of airway FBs and can be regarded as complementary to each other, and we emphasize the advantages for bronchoscopists to be familiar with both rigid and flexible techniques. Physicians should reperform bronchoscopy to identify any residual FB and pay special attention to abnormal chest images after removal of FB.

## Data Availability

The original contributions presented in the study are included in the article/**Supplementary Material**, further inquiries can be directed to the corresponding author.
