# Supplementary material for: A combination of flexible and rigid bronchoscopy in the successful removal of a residual fish bone from a peripheral bronchus: A case report
Source: Front Pediatr. 2023 Feb 21;11:1114043. doi: 10.3389/fped.2023.1114043 (PMC9989009; doi:10.3389/fped.2023.1114043)
Supplement: Supplementary file 1 [file Table1.docx]

***Supplementary Material***

| Table S1.Current research in the field of artifical intelligence (AI) about bronchoscopy procedures | | | |
| --- | --- | --- | --- |
| Authors | Application | Contribution of AI | References |
| Li et al., 2022 | AI-based diagnostic model for brochial lumen identification | Better recognize bronchial lumen, reduce difference in the operation levels of different bronchoscopists, imporve the quality of everday bronchoscopies | 1 |
| Zhang et al., 2021 | artifical intelligence technology for analyzing the pathology of hesperetin-derived small cell lung cancer under fiberoptic bronchoscopy | higher accuracy rate than that of lung biopsy (97.9% vs. 89%), low cost, short time | 2 |
| Asfahan et al., 2021 | application of AI during rapid on-site examination (rose)of Endobronchial ultrasound guided transbronchial needle aspiration (EBUS-TBNA) | high accuracy, substantial reduction in time and expense | 3 |
| Matava et al., 2020 | AI classify vocal cords and tracheal airway anatomy real-time during video laryngoscopy or bronchoscopy | improve airway managementand bronchoscopy by helping to identify key anatomy real time, theoretically be extended to the settings of airway pathology or airway management in the hands of experienced providers | 4 |
| Yoo et al., 2021 | video bronchoscopy with a decisiong supporting system using AI | distingusihing anatomical location among the carina and both main bronchi under random rotation and covering, designing a clinical support system with video bronchoscopy | 5 |
| Rodriguez-Diaz et al., 2021 | elastic scattering spectroscopy (ESS) coupled with machine learning, to distinguish lung lesions from the various nearby tissue types | improve reliability of pulmonary lesion targeting | 6 |

**Reference**

1. Li Y, Zheng X, Xie F, Ye L, Bignami E, Tandon YK, et al. Development and validation of the artificial intelligence (AI)-based diagnostic model for bronchial lumen identification. *Transl Lung Cancer Res*. (2022) 11:2261-2274. doi:10.21037/tlcr-22-761

2. Zhang X, Yu Z. Pathological analysis of hesperetin-derived small cell lung cancer by artificial intelligence technology under fiberoptic bronchoscopy. *Math Biosci Eng*. (2021) 18:8538-8558. doi:10.3934/mbe.2021423

3. Asfahan S, Elhence P, Dutt N, Niwas Jalandra R, Chauhan NK. Digital-Rapid On-site Examination in Endobronchial Ultrasound-Guided Transbronchial Needle Aspiration (DEBUT): a proof of concept study for the application of artificial intelligence in the bronchoscopy suite. *Eur Respir J*. (2021) 58:doi:10.1183/13993003.00915-2021

4. Matava C, Pankiv E, Raisbeck S, Caldeira M, Alam F. A Convolutional Neural Network for Real Time Classification, Identification, and Labelling of Vocal Cord and Tracheal Using Laryngoscopy and Bronchoscopy Video. *J Med Syst*. (2020) 44:44. doi:10.1007/s10916-019-1481-4

5. Yoo JY, Kang SY, Park JS, Cho YJ, Park SY, Yoon HI, et al. Deep learning for anatomical interpretation of video bronchoscopy images. *Sci Rep*. (2021) 11:23765. doi:10.1038/s41598-021-03219-6

6. Rodriguez-Diaz E, Kaanan S, Vanley C, Qureshi T, Bigio IJ. Toward optical spectroscopy-guided lung biopsy: Demonstration of tissue-type classification. *J Biophotonics*. (2021) 14:e202100132. doi:10.1002/jbio.202100132
